# Shortening of the Pittsburgh Sleep Quality Index Survey Using Factor Analysis

**DOI:** 10.1155/2018/9643937

**Published:** 2018-04-12

**Authors:** Oluremi A. Famodu, Makenzie L. Barr, Ida Holásková, Wenjun Zhou, Jesse S. Morrell, Sarah E. Colby, Melissa D. Olfert

**Affiliations:** ^1^Division of Animal and Nutritional Sciences, Davis College of Agriculture, Natural Resources, and Design, West Virginia University, Morgantown, WV, USA; ^2^Office of Statistics, West Virginia Agriculture and Forestry Experiment Station, West Virginia University, 4100 Agricultural Sciences Building, P.O. Box 6108, Morgantown, WV 26506-6108, USA; ^3^University of Tennessee, Knoxville, 916 Volunteer Boulevard, UT SMC 247, Knoxville, TN 37996, USA; ^4^University of New Hampshire, Kendall Hall, Room 115, 129 Main Street, Durham, NH 03824, USA; ^5^University of Tennessee, Knoxville, 1215 W. Cumberland Avenue, 229 Jessie Harris Building, Knoxville, TN 37996, USA

## Abstract

**Objective/Introduction:**

Lengthy surveys have the potential to burden users and can lead to inaccuracies. Conducting analyses to shorten existing validated surveys is beneficial. The objective, therefore, was to shorten the Pittsburgh Quality Sleep Index (PSQI) for young adults.

**Methods:**

PSQI data from 1246 college students were used. An exploratory factor analysis (FA) was utilized to shorten survey after dropping select items. Nonparametric correlation analysis (Spearman's rho) was conducted between the global sleep scores of the shortened and original surveys. Agreements tests (Kappa and McNemar's test) measured the agreement of the surveys and sensitivity and specificity were evaluated.

**Results:**

Six factors were examined using maximum likelihood factoring method, applying squared multiple correlations with Promax rotation to allow for correlated variables. FA with six factors explained 100% of shared variance based on eigenvalues and accounted for 61% of variability based on variables. The FA resulted in 13 selected questions (“shortPSQI”), corresponding to 5 of the 7 components of the original survey. High correlation was found between the global scores of the original survey and the “shortPSQI” (rho = 0.94, *p* < 0.001). When the global score was converted to the categorical variable of good or poor sleepers, the agreement test indicated strong agreement (Kappa 0.83, 95% CI 0.79–0.86, *p* < 0.0001).

**Conclusion:**

The validated, 19-item PSQI survey was shortened to 13 items. Tests of correlation and agreement indicate the “shortPSQI” may be an acceptable alternative to the original survey for young adults.

**Clinical Trial Registration:**

Data for this study was taken from the Get Fruved study, registered on October 21, 2016, on clinicaltrials.gov (NCT02941497).

## 1. Introduction

Research conducted in higher education institutions often relies on survey data to gather indicators of health (e.g., health behavior, health knowledge, and health practices). However, response burden is one potential issue that limits the amount and accuracy of data collected from survey distribution. Factors associated with response burden are questionnaire length, cognitive ability to complete the survey, and type of questionnaire interface [1]. In particular, lengthy surveys result in fewer respondents starting or completing questionnaires [2] and differences in processing questions near the end, which can lead to omitted information [3] and/or inconsistent answers [4]. Given the need to maintain high quality research, investigators have begun to explore ways to decrease response burden, including reducing the number of items in existing questionnaires [5]. Some studies take a smaller subset of questions from validated surveys with the intention of reducing response burden [6]. However, these truncated surveys must be revalidated before being used in research.

To assess the relationship between sleep and overall health, both quantitative and qualitative attributes of sleep are often investigated. College students are increasingly studied in the field of sleep due to their abnormal schedules and lower sleep quality [7]. College students' unhealthy lifestyle behaviors could be attributed to social and academic demands [7]. The adoption of unhealthy lifestyles impacts not only sleep, but also diet [8], physical activity [9], and mental health [10]. Often these global health-related behaviors are associated with lower academic success [10]. Although there are “gold standard” tools, such as polysomnography (PSG), to analyze sleep quality [11], these tools tend to be expensive and may not be feasible for use with larger sample sizes. Thus, most investigators use published survey scales related to sleep quantity and quality. The Pittsburgh Sleep Quality Index (PSQI) [12], a 19-item, self-rating tool, assesses sleep quality and discriminates between “good” and “poor sleepers.” The PSQI has been shown to have a high degree of internal consistency (Cronbach's alpha 0.83) and has been validated against clinical and laboratory diagnoses of “good” and “poor” sleepers following 18 months of field-testing [12]. However, the authors have found weak or inconsistent associations between the PSQI and PSG, which may be due to PSQI assessing habitual patterns of sleep and sleepiness, whereas PSG assesses sleep and sleepiness in one discreet occasion (Buysse 2000). Regardless, PSQI is a commonly used research tool that has shown stronger relationship to psychological symptoms and stress compared to other subjective sleep tools [13] (i.e., Epworth Sleepiness Scale) [14]. Therefore, it can be further examined to see whether shortening maintains adequate statistical validity with the original tool. This in turn would reduce participant response burden associated with using this sleep assessment tool. Therefore, the present study used a factor analysis to shorten the 19-item, validated PSQI survey using a large sample of college students and compared the calculated variables of the shortened version to the original version. The hypothesis is that a shortened tool will be generated that will show high agreement with the original survey.

## 2. Methods

### 2.1. Sample

College freshmen across eight universities (West Virginia University, University of Tennessee, University of Maine, Syracuse University, University of Florida, Kansas State University, Auburn University, and South Dakota State University) were recruited in the fall of 2015 to participate in the* Get Fruved* study.* Get Fruved* is a USDA-funded project designed to prevent the development of overweight and obesity among older adolescents by developing a peer-led, interactive, social marketing intervention focusing on dietary quality, increased physical activity, and stress management along with environmental support. All incoming first year students were invited to participate. To be eligible to participate, the students had to be consuming less than the recommended intakes for fruits or vegetables and meet one additional criteria (including being first-generation college student, being of racial/ethnic minority, being overweight or obese, having a parent who is overweight or obese, or coming from a lower affluent household). A total of 1374 college students were interested and eligible to be in the study; however only 1246 students completed the PSQI survey in its entirety. Each university's Institutional Review Board approved the study and every participant signed a consent form. Participants then completed a behavioral survey with questions on many different health-related areas, including fruit and vegetable intake (NCI Fruit and Vegetable Screener) [15], stress (Cohen's Perceived Stress Scale) [16], sleep behavior (PSQI) [12], physical activity (International Physical Activity Questionnaire) [17], and other health measurements.

### 2.2. Original PSQI Measuring Instrument

**Figure 1:**
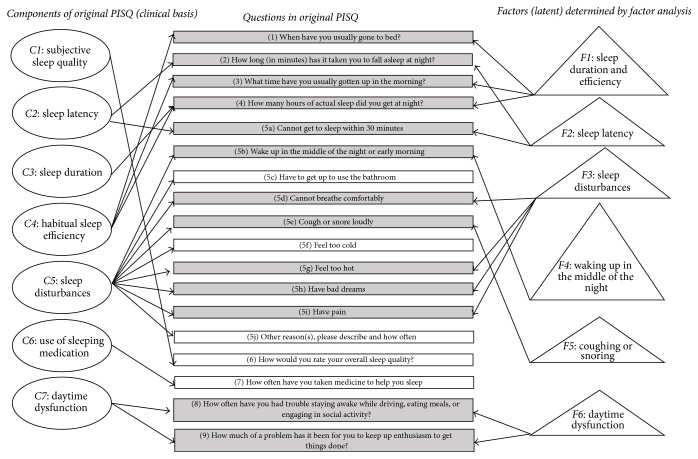
Graphical representation of seven* clinically determined* components (ovals) of original PSQI and their corresponding questions (rectangles) as well as the* factor analysis-based* six factors (triangles).

In the sleep section of the survey, students were asked to recall their sleep habits over the past month to answer 19 individual questions related to seven main components: sleep quality, sleep latency, sleep duration, habitual sleep efficiency, sleep disturbance, use of sleep medication, and daytime dysfunction. PSQI is described in more detail by Buysse et al. [12]. Middle panel of Figure 1 lists the 19 questions, organized in 18 items (Question (5j) had a subquestion) of the original scale and left panel of ovals lists the seven components to which each item belonged. Following the protocols established by Buysse et al. [12], including minor preprocessing of total number of hours in bed and habitual sleep efficiency, all components received a score based on a Likert scale of 0–3, where a score of 0 reflected the positive extreme (“not during the past month”) and score of 3 reflected a negative extreme. A global PSQI score was generated for each participant after summing the seven components (all weighted equally on a 0–3 scale), with a final range of 0–21 where higher scores indicated worse sleep quality. A global PSQI score > 5 was indicative of poor sleep quality, relative to clinical and laboratory measures.

### 2.3. Statistical Test for Developing “shortPSQI”

In order to shorten the original 19-question PSQI survey, an exploratory factor analysis (FA) with Promax rotation was conducted on all 17 items, using varying number of factors (5–8), and after minimal preprocessing of some variables. This included conversion and calculation of differences between time entered for going to bed (Question (1)) and time entered for getting up (Question (3)), as well as scoring Question (5j) (other reasons for sleep disturbances and how often) into one item, thus reducing the items from 19 to 17 entered into the FA, listed in Table 4. Promax, a method used in FA, represents oblique rotation and allows for correlation among the variables.

Based upon results of FA, three methods of scoring calculations were done. One method utilized the original components of the survey (“shortPSQI”) and the other two used new components based off the generated factors. All scoring methods were validated and assessed for agreement with original PSQI.

### 2.4. Statistical Test for Testing Validity for Shortened Survey

For validation of the FA, a nonparametric correlation (Spearman's rho) was used to measure the consistency between the global sleep score of the shortened survey and the global score of the original survey. In addition, Kappa and McNemar's tests were used to measure the agreement of the survey in detecting number of “good” and “poor” sleepers. Efficacy tests of specificity and sensitivity were also conducted to measure the proportion of “poor” and “good” sleepers detected by the shortened survey to the original questionnaire. A Receiver Operating Characteristic (ROC) curve was constructed to ensure that the most acceptable degree of sensitivity and specificity was maintained in order to validate the shortened survey cut-off score that distinguished between “poor” and “good” sleepers.

All statistical analyses were completed using JMP and SAS software (JMP®, Version Pro 12, SAS Institute Inc., Cary, NC, Copyright ©2015; SAS®, Version 9.3, SAS Institute Inc., Cary, NC, Copyright ©2002–2010). Significance criterion alpha for all tests was 0.05.

## 3. Results

### 3.1. Developing “shortPSQI” Survey

Rotated factor loadings from the FA on *n* = 1246 subjects data using the original 19 questions (17 items) are listed in Table 4. Six factors (Factors 1–6) were extracted using Promax rotation, which was done after orthogonal (Varimax) rotation using proportion of shared variance criteria based on eigenvalues (100%), scree test, and interpretability. In addition, decision was made for maintaining those loading values exceeding 0.3 on the rotated loading. Using such criteria,* Factor 1* was represented by the sleep duration and length of time in bed, calculated later into the habitual sleep efficiency (hours of sleep/hours in bed) *∗* 100 before final scoring. (Q2) and (Q5a) both loaded strongly on* Factor 2* and relate to how long it takes a person to fall asleep. (Q5d), (Q5g), (Q5h), and (Q5i) loaded on* Factor 3* that may be collectively named as sleep disturbances due to lack of comfort including pain, problems in breathing, being too hot, and having bad dreams. Waking up in the middle of the night or early was slightly correlated to going to bathroom, but was found unusually solitary loading on* Factor 4*. Likewise, coughing and snoring loudly (Q5e) loaded in isolation onto* Factor 5*. Daytime dysfunction was represented by the last two questions that inquired in more detail about troubles staying awake while driving or eating, or during social activity, in addition to problem with enthusiasm to get things done, both loading heavily on* Factor 6*.

From this interpretation, 13 questions were selected for the “shortPSQI” and they corresponded to 5 of the 7 components of the original survey (sleep duration, sleep latency, sleep efficiency, sleep disturbances, and daytime dysfunction) (Figure 1, items in grey). The omitted questions of all loadings less than 0.30 belonged to using bathroom (Q5c), feeling cold (Q5f), other sleep disturbances not mentioned (Q5j), overall sleep quality (Q6), and frequency of using medicine to help fall asleep (Q7).

### 3.2. Scoring of “shortPSQI”

Questions with a rotated factor loading of 0.30 or higher were first organized and evaluated as assigned to the original clinically based components by Buysse et al. [12]. These components included C2: sleep latency, C3: sleep duration, C4: habitual sleep efficiency, C5: sleep disturbances, and C7: daytime dysfunction. If certain questions were omitted due to very low or negative values (loadings) and contributed to certain component score (i.e., sleep disturbances), the summation of all questions attained a smaller maximum as in the original. These final scores for the whole component were proportioned to fit a range of 0 to 3. To illustrate the algorithm, the “sleep disturbance” component in the original survey contained nine questions. Each of them was first scored on a 0–3 Likert scale and then added together for a total score between 0 and 27 for this component [12]. In order for this sum to be on the final scale of 0–3, the total score was divided into four corresponding subsets: zero and the remaining into 3 equal-distance portions: ≥1 and ≤9 (given a score of 1), >9 and ≤18 (given a score of 2), and >18 (given a score of 3), proportional to the max value 27. Having a zero in this component meant the individual had none of these disturbances in the past month, and three (>18) indicated the individual experienced a high amount of disturbances over the past month. The same method was applied to the “shortPSQI” where we first scored our six of the nine remaining questions in this component ((Q5b) wake up in the middle of the night or early morning; (Q5d) can't breathe comfortably; (Q5e) cough or snore loudly; (Q5g) feel too hot; (Q5h) have bad dreams; and (Q5i) have pain) on a 0–3 scale and then summed the questions' scores to generate a total between 0 and 18. By dividing this into three equal subsets and a zero category, we derived the following: 0, ≥1 and ≤6 corresponding to 1, >6 and ≤12 corresponding to 2, and >12 corresponding to final component score of 3 (see Scoring “shortPSQI” in Appendix).

Lastly, because the questions of overall sleep quality and sleep medications were not included in the loading plot, these two components were eliminated from the “shortPSQI” leaving 5 remaining components. All 5 remaining components scored between 0 and 3 points; this resulted in a global “shortPSQI” ranging between 0 and 15, instead of the original 0 and 21. In order to create a categorical variable of “good” and “poor” sleepers, a cross multiplication approach was used with the cut-off threshold of 5 from the original scale of 0 to 21 [12]. Using this method, a global PSQI score > 3.57 on the “shortPSQI” was indicative of a poor-quality sleeper. However, agreement analysis, as well as measures of sensitivity and specificity, yielded better estimation (closer to original PSQI) using a cut-off > 4 to detect poor-sleeper on the “shortPSQI.”

### 3.3. Validity Testing of “shortPSQI”

**Figure 2:**
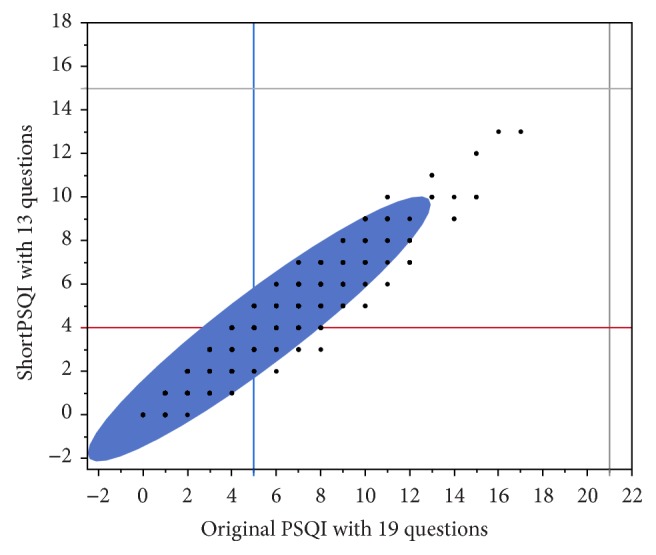
Spearman correlation of 0.94 (*p* < 0.0001) was detected between scores of original PSQI and shortened survey. Grey lines represent maximal possible global score in original PSQI (21) and “shortPSQI” (15). Blue vertical line represents cut-off 5 on PSQI; all who had more than 5 were assigned as poor sleepers. Red horizontal line represents cut-off point of 4 in shortPSQI; all students above the line were defined as poor sleepers.

The average global score of the original survey was 5.23 (SD 2.54) (Table 1) in 1246 college students who completed the entire survey (Table 2). The “shortPSQI” averaged 4.00 (SD 2.03) using the same data. Correlations between the original survey and the “shortPSQI” are depicted in Figure 1. The “shortPSQI” showed a significant correlation with the original global scale (*ρ* = 0.94, *p* < 0.001) (Figure 2).

After the “good” and “poor” sleepers were calculated in each of the surveys for various cut-off points of the newly developed scores, Kappa showed significant strong agreement with the “shortPSQI” at a cut-off of 4 and original survey cut-off of 5 (Kappa 0.83, *p* < 0.001) (Table 2). However, the test statistics also showed significance (*p* < 0.001) in McNemar's test. McNemar's test for two-by-two tables is equivalent to Bowker's test of symmetry of disagreement. Significant value may be indicating lack of agreement or that the “false” counts (false negative and false positive) are not equally balanced. Considering the data in Table 2, the “shortPSQI” may be slightly underestimating number of poor sleepers. When looking at sensitivity of the “shortPSQI” in detecting the proportion of “poor” sleepers of those identified as “poor” sleepers by the original, the “shortPSQI” correctly identified 83.4% (423/507). Specificity (the proportion of “good” sleepers detected by the shortened method of the original “good” sleepers) was also high, where the “shortPSQI” correctly identified 97.4% (720/739).

As an additional measure of comparison to the original survey, we took the overall sleep quality score (C1) and evaluated it against those identified as “poor” and “good” sleepers of the “shortPSQI” global score. The Likert scale values on C1 from 0 to 3 corresponded to “very good, fairly good, fairly bad, and very bad,” respectively. When the responses were categorized into 2 categories, good (0, 1) and poor (2, 3), there were 15.25% of students (190/1246) who classified themselves as poor sleepers. The original PSQI identified 40.4% poor sleepers from the same population (507/1246), and the “shortPSQI” identified 35.5% poor sleepers (442/1246), using cut-off 4.

**Figure 3:**
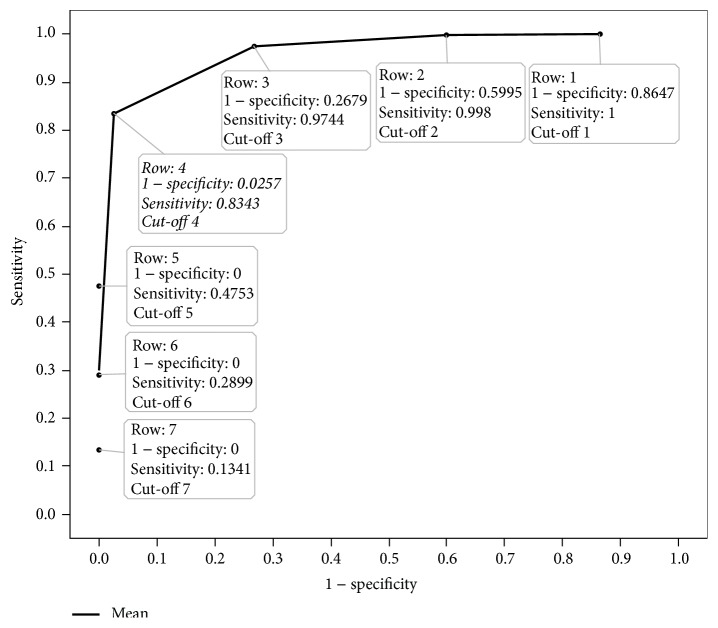
Receiver Operating Characteristic (ROC) curve. The ROC curve is generated from seven different cut-off points of global scores on “shortPSQI” survey, using sensitivity (true positive) and 1 − specificity (specificity = true negative; 1 − specificity = false negative) with respect to cut-off 5 on original PSQI questionnaire. The cut-off of >4 on the global score of the “shortPSQI” demonstrates the highest sensitivity and specificity compared to the other cut-off values.

Figure 3 presents the ROC curve based off 5 possible cut-off PSQI scores in determining “poor” versus “good” sleepers for the “shortPSQI” and their corresponding sensitivities and specificities (Table 3). The cut-off 4, closest upper integer to what we derived mathematically (see Scoring of “shortPSQI”), demonstrates the highest sensitivity and specificity compared to the other cut-off points.

### 3.4. Additional Scoring Methods for Shortened Survey

Additional methods of scoring the shortened survey were undertaken utilizing the 6 factors and generating new components (Figure 1, triangles). Both of these additional methods overestimated the number of poor sleepers and did not yield favorable validation proportions (sensitivity and specificity, data not shown). More information on the scoring methods can be found in Appendix NCT02941497.

## 4. Discussion

Lengthy surveys are likely to overburden participants and increase inaccuracies if participants rush or omit information to finish. Shortening existing validated surveys may reduce the time required for participants to complete these surveys allowing for improved data collection efforts. The results of this study are encouraging for downsizing the 19-item PQSI survey to assess sleep habits and sleep quality in young adults. Questionnaire length was reduced by over one-third with minimal loss in original survey components. The 13-item “shortPSQI” reached similar conclusions based upon the global score and categorical variable compared to the original survey. Thus, using three honest assessments of validity (correlation, Kappa, and sensitivity/specificity), the shortened version of the questionnaire represents a plausible alternative to the original PSQI.

While scoring the “shortPSQI,” a concern arose due to a latent factor that excluded more than 33% of the questions about individual sleep disturbances. This could be the reason why we observed discordance by McNemar's test because we detected less “poor” sleepers than the original survey (35.5% and 40.1%, resp.). We believe this survey is specific to the young adult population where some of the clinical disturbances (i.e., sleep medications) of sleep may not be as high in this population; therefore this short survey may be more accurate to sleep problems associated with this population. If choosing between the assessment tools, it is important to consider the extent of sleep quality analysis the researcher hopes to achieve from their sample population. The 19-item survey defines more levels of sleep disturbances than does the 13-item survey (“shortPSQI”). However, the shorter version is attractive for larger, younger, population samples with time constraints or those who wish to administer surveys with many items.

In conducting the analyses to shorten the original PSQI survey, there were some methodological questions that arose. First, it should be noted that the analyses and validation methods used to derive the conclusions of the 13-item PSQI survey were based on the data from the 19-item PSQI distributed to college students. We assume that the same or better results will be obtained when the “shortPSQI” is administered alone. The FA excluded the “sleep quality” and “sleep medication” component in the shortened version which is also a concern. Although it was adjusted for in the global PSQI score of the 13-item questionnaire, there is still an issue of whether these components are needed to measure overall quality of sleep in a participant. However, the authors agree that reliability (or consistency) of the 13-item questionnaire would have been ideal to measure by administering the same survey to the same group of participants in a short duration, given that sleep patterns did not change. Yet, this was not feasible for a large sample size where data was contributed by multiple institutions across the United States. Lastly, although we feel like this was a large enough dataset to be able to decrease the number of items in the original survey, this survey was only distributed to young adults attending college; therefore the “shortPSQI” may not be generalizable to other samples. Thus, further research is encouraged in other populations.

## 5. Conclusion

In conclusion, this is the first study that shortened the validated, 19-item PSQI survey to a 13-item survey (“shortPSQI”) using a factor analysis on responses from over one thousand students. Testing demonstrated that the “shortPSQI” correlated and agreed with the original survey and could potentially be used to derive the same outcomes with reduced respondent burden. Further validation would include administering the “shortPSQI” and original survey to a new sample, with the addition of validating it against “gold” measures of sleep like PSG.

## Figures and Tables

**Table 1:** Global PSQI score of the original and the “shortPSQI.”

Survey (*n* = 1246)	Mean	SD
Original 19-item PSQI	5.23	2.54
Short 13-item PSQI	4.00	2.03

*Note*. SD: standard deviation.

**Table 2:** Agreements tests between “good” and “poor” sleeper scored by original and “shortPSQI.”

Original survey	“shortPSQI”		Total
	*Poor sleepers*	*Good sleepers*	
*Poor sleepers*	423	84	507
*Good sleepers*	19	720	739
Total	442	804	1246

Kappa value 0.83 indicates an agreement (*p* < 0.001) between original and “shortPSQI,” based on a cut-off of 5 and 4 in PISQ and “shortPSQI,” respectively; McNemar's test indicated a lack of agreement (*p* < 0.0001) or, rather, a lack of symmetry of false negative and false positive proportions. Based on the analyses, all global values greater than 4 in “shortPSQI” total score are detecting poor sleepers.

**Table 3:** Specificity and sensitivity of different cut-off values for “good” and “poor” sleeper scored by original and “shortPSQI.”

Cut-off Value^a^ (shortPSQI)	Sensitivity^b^	Specificity^c^
1	507/507 = 100%	100/739 = 13.53%
2	506/507 = 99.8%	296/739 = 40.05%
3	494/507 = 97.44%	541/739 = 73.21%
4	423/507 = 83.43%	720/739 = 97.43
5	241/507 = 47.53%	739/739 = 100%
6	147/507 = 28.99%	739/739 = 100%
7	68/507 = 13.41%	739/739 = 100%

^a^“shortPSQI” cumulative score greater than this cut-off indicates “poor” sleeper. ^b^Detecting the proportion of “poor” sleepers determined by “shortPSQI” out of original survey “poor” sleepers. ^c^Detecting the proportion of “good” sleepers determined by “shortPSQI” out of original survey “good” sleepers.

**Table 4:** Rotated factor loading in factor analysis Using Promax rotation.

PSQI and Fruved questions	*Factor 1*	*Factor 2*	*Factor 3*	*Factor 4*	*Factor 5*	*Factor 6*
*(Q2) How long has it taken you to fall asleep?*	0.034972	*0.787579*	0.031071	−0.06686	−0.02542	−0.0754
*(Q3)-(Q1) Hours in bed?*	*0.836975*	0.098359	0.011752	0.021052	−0.02628	0.020742
*(Q4) Actual sleep hours?*	*0.846725*	−0.06632	−0.02319	0.032104	0.012722	5.83*E* − 05
*(Q5a) Can't get to sleep within 30 min?*	0.002007	*0.800826*	−0.00747	0.056409	0.03448	0.008737
*(Q5b) Wake up in the middle of the night or early?*	−0.0202	−0.01773	−0.01025	*1.019651*	−0.01481	−0.04104
(Q5c) Get up to use the bathroom?	0.123561	−0.00289	0.132605	0.285131	0.032987	0.020361
*(Q5d) Can't breathe comfortably?*	−0.02466	0.024227	*0.39047*	−0.0176	0.25699	−0.03796
*(Q5e) Cough or snore loudly?*	−0.00746	0.000919	−0.01287	0.003121	*1.003774*	0.001288
(Q5f) Do you feel too cold?	0.022562	−0.00962	0.252864	0.095624	0.105187	0.123154
*(Q5g) Do you feel too hot?*	0.090277	0.038497	*0.378038*	0.124697	0.034749	0.032433
*(Q5h) Do you have bad dreams?*	−0.00799	−0.00031	*0.434513*	0.159701	−0.03889	0.001685
*(Q5i) Do you have a pain?*	−0.07019	0.000458	*0.575351*	−0.00287	−0.0191	−0.02643
(Q5j) Other reasons and how often?	−0.03039	0.055302	0.234025	−0.06613	−0.06605	0.067663
(Q6) Overall sleep quality	−0.21386	0.275513	0.003293	0.135137	−0.00909	0.261002
(Q7) How often do you use medicine to help you sleep?	0.039606	0.181535	0.147002	−0.04779	0.01667	0.082251
*(Q8) Do you have trouble staying awake during the day?*	−0.00298	−0.06265	0.035186	−0.03087	0.025594	*0.560634*
*(Q9) Do you have problem keeping enthusiasm and getting things done?*	0.033092	0.014256	0.021721	−0.01387	−0.02722	*0.657612*

(Q3)–(Q1) represents Q1 value being subtracted from Q3, that is, (Q3 in military time) + (24–Q1 in military time).

## Data Availability

The datasets used and analyzed during the current study are available from the corresponding author on reasonable request.

## Appendix

### A. Scoring “shortPSQI”

  Name —
  ID # —
  Date —
  Age —

*Instructions*. The following questions relate to your usual sleep habits during the past month only. Your answers should indicate the most accurate reply for the majority of days and nights in the past month.

Please answer all questions.

1. During the past month, when have you usually gone to bed?
    —
2. During the past month, how long (in minutes) has it taken you to fall asleep each night?
    —
3. During the past month, when have you usually gotten up in the morning?
    —
4. During the past month, how many actual hours of sleep did you get at night? (This may be different than the number of hours you spend in bed.)
    —

For each of the remaining questions, check the one best response. Please answer all questions.

(5) During the past month, how often have you had trouble sleeping because you…
  - Cannot get to sleep within 30 minutes
      Not during the past month —
      Less than once a week —
      Once or twice a week —
      Three or more times a week —
  - Wake up in the middle of the night or early morning
      Not during the past month —
      Less than once a week —
      Once or twice a week —
      Three or more times a week —
  - Cannot breathe comfortably
      Not during the past month —
      Less than once a week —
      Once or twice a week —
      Three or more times a week —
  - Cough or snore loudly
      Not during the past month —
      Less than once a week —
      Once or twice a week —
    Three or more times a week —
  - Feel too hot
      Not during the past month —
      Less than once a week —
      Once or twice a week —
      Three or more times a week —
  - Have bad dreams
      Not during the past month —
      Less than once a week —
      Once or twice a week —
      Three or more times a week —
  - Have pain
      Not during the past month —
      Less than once a week —
      Once or twice a week —
      Three or more times a week —
(6) During the past month, how often have you had trouble staying awake while driving, eating meals, or engaging in social activity?
    Not during the past month —
    Less than once a week —
    Once or twice a week —
    Three or more times a week —
(7) During the past month, how much of a problem has it been for you to keep up enthusiasm to get things done?
    Not during the past month —
    Less than once a week —
    Once or twice a week —
    Three or more times a week —

*Scoring*

*Component 1: sleep latency*
    C1 —
    (#2) Score (<15 (0), 16–30 min (1), 31–60 min (2), >60 min (3)) + (#5a) Score (if sum is equal 0 = 0; 1-2 = 1; 3-4 = 2; 5-6 = 3)
*Component 2: sleep duration*
    C2 —
    (#4) Score (>7 (0), 6-7 (1), 5-6 (2), <5 (3))
*Component 3: sleep efficiency*
    C3 —
    (total # of hours asleep)/(total # of hours in bed) × 100; >85% = 0, 75–84% = 1, 65–74% = 2, <65% = 3
*Component 4: sleep disturbances*
    C4 —
    Examine questions (5b) to (5g) and assign scores for each questions as follows
    *Response*:* Score*
      Not during the past month: 0
      Less than once a week: 1
      Once or twice a week: 2
      Three or more times a week: 3
    # sum of scores (5b) to (5g) (0 = 0, ≥1 ≤ 6 = 1; >6 ≤ 12 = 2; >12 = 3)
*Component 5: daytime dysfunction*
    C5 —
    (#6) + (#7) Score (0 = 0; 1-2 = 1; 3-4 = 2; 5-6 = 3)

*Add the five component scores together…Global PSQI* —

*A total score of greater than “4” is indicative of poor sleep quality*

#### A.1. Rotated Factor Loading Using Promax Rotation (See Table 4)

The goal of factor analysis was to understand if there are underlying factors encompassing variables measured by PSQI questionnaire and determine which variables (questions) are essential contributors to these factors. Six factors (Factors 1–6) were extracted using Promax rotation, which is done after orthogonal (Varimax) rotation using proportion of shared variance criteria based on eigenvalues, scree test, and interpretability. Promax represents oblique rotation and allows for correlation among the variables.* Factor 1* represents the sleep duration and length of time in bed, calculated later into the habitual sleep efficiency (hours of sleep/hours in bed) *∗* 100 before scoring. (Q2) and (Q5a) both load strongly on* Factor 2* and both relate to how long it takes a person to fall asleep. (Q5d), (Q5g), (Q5h), and (Q5i) load on* Factor 3* that may be collectively named as sleep disturbances due to lack of comfort including pain, problems breathing, being too hot, and having bad dreams. Waking up in the middle of the night or early was slightly correlated to going to bathroom but was found unusually solitary loading on* Factor 4*. Likewise, coughing and snoring loudly (Q5e) loaded alone in isolation onto* Factor 5*. Daytime dysfunction was represented by the last two questions that inquired in more detail about troubles staying awake while driving or eating or during social activity, in addition to problem with enthusiasm to get things done, both loading heavily on* Factor 6*.

#### A.2. Scoring of Additional Methods

Additional two methods also utilized the shorter survey (13 items), but the 6 factors, detected by FA, were considered independent of each other and scored independently of groupings from the original seven components. Namely, six FA-identified factors listed in Figure 1 (right panel of triangles), such as F1, sleep duration and efficiency; F2, sleep latency; F3, sleep disturbances; F4, waking up in the middle of the night; F5, coughing and snoring; and F6, daytime dysfunction, were scored in a similar manner as above (Likert scale: 0, 1, 2, and 3) but with using 13 items, 6 factors in a role of six components, and then scored for global score. The first of the two methods of scoring of the components used equal weight for each component and simply summed up the scores of 6 components for total of 18 points with cut-off of 4.28. This method identified 53.9% of poor sleepers (672/1246). In the second method, specific weight for each component was determined from multivariate regression expressed as a proportion of total variability (PV) from sums of squares (SS) contributed by each component to global PSQI and used as a multiplication coefficient. For F1, sleep duration and efficiency PV = 0.306; F2, sleep latency PV = 0.352; F3, sleep disturbances PV = 0.015; F4, waking up in the middle of the night PV = 0.013; F5, coughing and snoring PV = 0.009; and F6, daytime dysfunction PV = 0.306 were used. Then the products of the scores and their respective coefficients were added for maximal total of 3 and cut-off 0.714 was determined. This method identified 48.8% of poor sleepers (608/1246).
